# Supplementary material for: Association between weight-adjusted waist index and periodontitis: A population-based study
Source: PLoS One. 2024 Jun 6;19(6):e0305010. doi: 10.1371/journal.pone.0305010 (PMC11156281; doi:10.1371/journal.pone.0305010)
Supplement: S2 Table — (DOCX) [file pone.0305010.s002.docx]

**S2 Table.** The associations between weight-adjusted waist index and severity of periodontitis.

| **Exposure** | **Model 1 [OR (95% CI)]** | **Model 2 [OR (95% CI)]** | **Model 3 [OR (95% CI)]** |
| --- | --- | --- | --- |
| No periodontitis | reference | reference | reference |
| Mild | 1.08 (0.96, 1.22) | 1.27 (1.11, 1.46) | 1.12 (1.04, 1.20) |
| Moderate | 1.59 (1.50, 1.68) | 1.42 (1.33, 1.52) | 1.23 (1.15, 1.32) |
| Severe | 1.33 (1.22, 1.46) | 1.31 (1.18, 1.46) | 1.12 (1.01, 1.25) |

Model 1: no covariates were adjusted. Model 2: age, gender, and race were adjusted. Model 3: age, gender, race, education level, PIR, smoking, alcohol drinking, diabetes, triglycerides, and high-density lipoprotein cholesterol were adjusted.
