# Supplementary material for: Association between weight-adjusted waist index and periodontitis: A population-based study
Source: PLoS One. 2024 Jun 6;19(6):e0305010. doi: 10.1371/journal.pone.0305010 (PMC11156281; doi:10.1371/journal.pone.0305010)
Supplement: S3 Table — (DOCX) [file pone.0305010.s003.docx]

**S3 Table.** Threshold effect analysis of WWI on periodontitis using two-piecewise linear regression model.

|  | Fitting by the standard linear model | Fitting by the two-piecewise linear model | | | |
| --- | --- | --- | --- | --- | --- |
|  |  | Inflection point (K) | <K-segment effect | >K-segment effect | Log likelihood ratio |
| WWI | 1.20 (1.12, 1.28)  <0.0001 | 11.99 | 1.11 (1.03, 1.20)  0.0096 | 1.97 (1.48, 2.62)  <0.0001 | <0.001 |

Age, gender, race, education level, PIR, smoking, alcohol drinking, diabetes, triglycerides, and high-density lipoprotein cholesterol were adjusted.
